# mGluR5 regulated proliferation of neural stem cells after hypoxia with activation of MAPK signaling pathway

**DOI:** 10.1186/1750-1326-7-S1-S18

**Published:** 2012-02-07

**Authors:** Lingyu Zhao, Qian Jiao, Xinlin Chen, Pengbo Yang, Bingqiao Zhao, Ping Zheng, Yong Liu

**Affiliations:** 1 Institute of Neurobiology, Xi'an Jiaotong University College of Medicine, 710061, China; 2 The State Key Laboratory of Medical Neurobiology, shanghai, 200032, China

## Background

Hypoxia/ischemia induces the neural stem cells (NSCs) proliferation in mammalian brain; but the mechanisms remain unknown.

## Methods

In this study, we investigated the effects of metabotropic glutamate receptor 5 (mGluR5) on NSC proliferation under hypoxia by 3-(4,5-dimethylthiazol-2-yl)-2,5-diphenyltetrazolium bromide (MTT) assay, diameter measurement of neurospheres, bromodeoxyuridine (BrdU) incorporation assay and cell cycle analysis. The cell death of NSCs was evaluated by terminal dUTP nick-end labeling (TUNEL) assay and Hoechst staining. The expression of cyclin D1 and the activation of mitogen-activated protein kinases (MAPKs) signaling pathway were analyzed by immunoblotting assay.

## Results

The results showed that hypoxia promoted the mGluR5 expression on NSCs. Under hypoxia, mGluR5 agonist DHPG and CHPG significantly increased NSC proliferation in cell activity, diameter of neurospheres, bromodeoxyuridine (BrdU) incorporation and cell division, and expression of cyclin D1 with decreasing of cell death. mGluR5 siRNA and antagonist MPEP decreased the NSC proliferation and expression of cyclin D1 with increasing of cell death. Phosphorylated JNK and ERK increased with the proliferation of NSCs after mGluR5 agonist DHPG and CHPG treatment under hypoxia, while p-p38 level decreased.

## Conclusions

These results demonstrated that the expression of mGluR5 was upregulated during the proliferation of NSCs stimulated by hypoxia in vitro. The activation of ERK and JNK signaling pathway and the expression of cyclin D1 were increased in the process. These finding suggesting the involvement of mGluR5 in NSC proliferation and providing a target molecule in neural repair after ischemia/hypoxia injury of CNS.

